# Causal relationships between immune cells and common urinary system tumors: A bidirectional analysis of Mendelian randomization

**DOI:** 10.1097/MD.0000000000044297

**Published:** 2025-09-12

**Authors:** Zhaohui Liu, Jingjing Tu, Jiaye Long, Lijun Guo

**Affiliations:** a Department of Urology, Inner Mongolia Forestry General Hospital, The Second Clinical Medical School of Inner Mongolia Minzu University, Yakeshi, Inner Mongolia, China; b Department of Gastrointestinal Surgery, Inner Mongolia Forestry General Hospital, The Second Clinical Medical School of Inner Mongolia Minzu University, Yakeshi, Inner Mongolia, China; c Department of Interventional Radiology, Inner Mongolia Forestry General Hospital, The Second Clinical Medical School of Inner Mongolia Minzu University, Yakeshi, Inner Mongolia, China.

**Keywords:** bladder cancer, immune cells, kidney cancer, Mendelian randomization, prostate cancer, urinary system tumors

## Abstract

We conducted research on the causal relationships between human immune cells and common urinary system tumors. This study conducted 2-sample Mendelian randomization analyses to determine the causal relationships between immune cell traits and the risk of kidney, bladder, and prostate cancers (PCs). Sensitivity analyses were used to validate the robustness of the results, focusing on pleiotropy and heterogeneity. These findings may inform early diagnosis and personalized immunotherapy for urinary system tumors. Our study identified 12 immune cell phenotypes associated with kidney cancer, including CD127− CD8br absolute cell (AC; odds ratio [OR] = 1.205, 95% confidence interval [CI]: 1.099–1.320, *P* = 6.73 × 10^−5^); CD25 on CD39+ activated regulatory T cell (OR = 1.136, 95% CI: 1.037–1.244, *P* = .006); CD4 on TD CD4+ (OR = 1.102, 95% CI: 1.027–1.182, *P* = .007); immunoglobulin D (IgD)+ CD38−% lymphocyte (OR = 1.101, 95% CI: 1.031–1.177, *P* = .004); CD20 on IgD− CD38br (OR = 1.067, 95% CI: 1.017–1.120, *P* = .008); CD25 on B cell (OR = 1.067, 95% CI: 1.022–1.114, *P* = .004); HLA DR on B cell (OR = 0.929, 95% CI: 0.882–0.979, *P* = .006); HLA DR on CD33dim HLA DR+ CD11b− (OR = 0.924, 95% CI: 0.874–0.977, *P* = .005); HLA DR on CD14+ CD16+ monocyte (OR = 0.947, 95% CI: 0.916–0.980, *P* = .002); CD62L− plasmacytoid DC %DC (OR = 0.906, 95% CI: 0.850–0.965, *P* = .002); CD11c on myeloid DC (OR = 0.904, 95% CI: 0.853–0.959, *P* = 7.38 × 10^−4^); CD11c on CD62L+ myeloid DC (OR = 0.930, 95% CI: 0.881–0.981, *P* = .008). Four immune cell phenotypes were associated with bladder cancer, including CD38dim% lymphocyte (OR = 1.081, 95% CI: 1.020–1.145, *P* = .009); HLA DR+ CD8br AC (OR = 0.940, 95% CI: 0.897–0.985, *P* = .009); IgD on unsw mem (OR = 0.908, 95% CI: 0.856–0.963, *P* = .001). In the conventional dendritic cell group, FSC-A on granulocyte (OR = 0.897, 95% CI: 0.836–0.963, *P* = .003). Six immune cell phenotypes were associated with PC, including CD19 on IgD− CD38− (OR = 1.080, 95% CI: 1.033–1.129, *P* = 6.21 × 10^−4^); CD27 on CD20− (OR = 1.040, 95% CI: 1.010–1.072, *P* = .009); CD86+ plasmacytoid DC %DC (OR = 1.053, 95% CI: 1.013–1.094, *P* = .009); CD25 on IgD+ CD38− (OR = 0.974, 95% CI: 0.960–0.988, *P* = 2.59 × 10^−4^); CD25hi CD45RA+ CD4 not regulatory T cell AC (OR = 0.962, 95% CI: 0.937–0.989, *P* = .006); CD127 on CD28− CD8br (OR = 0.952, 95% CI: 0.920–0.984, *P* = .004). Our study, utilizing Mendelian randomization genetic methods, has demonstrated the causal associations between immune cell phenotypes and kidney cancer, bladder cancer, and PC, providing guidance for future clinical diagnosis and treatment of these 3 malignant tumors of the urinary system.

## 1. Introduction

In 2020, global cancer statistics indicated approximately 19.3 million new cases and claimed the lives of around 10 million individuals. Among the 36 types of cancer, the incidence rate of new prostate cancer (PC), bladder cancer and kidney cancer ranked the 3rd, 11th and 16th among malignant tumors respectively, the mortality rate ranks 8th, 14th, and 16th respectively.^[1]^ Malignant tumors of the urinary system mentioned above have the highest incidence rate and mortality. Despite significant advancements in diagnosing and treating prostate, bladder, and kidney cancers, the outlook remains suboptimal as many patients are diagnosed in advanced stages, precluding surgical options.

**Figure 1.**
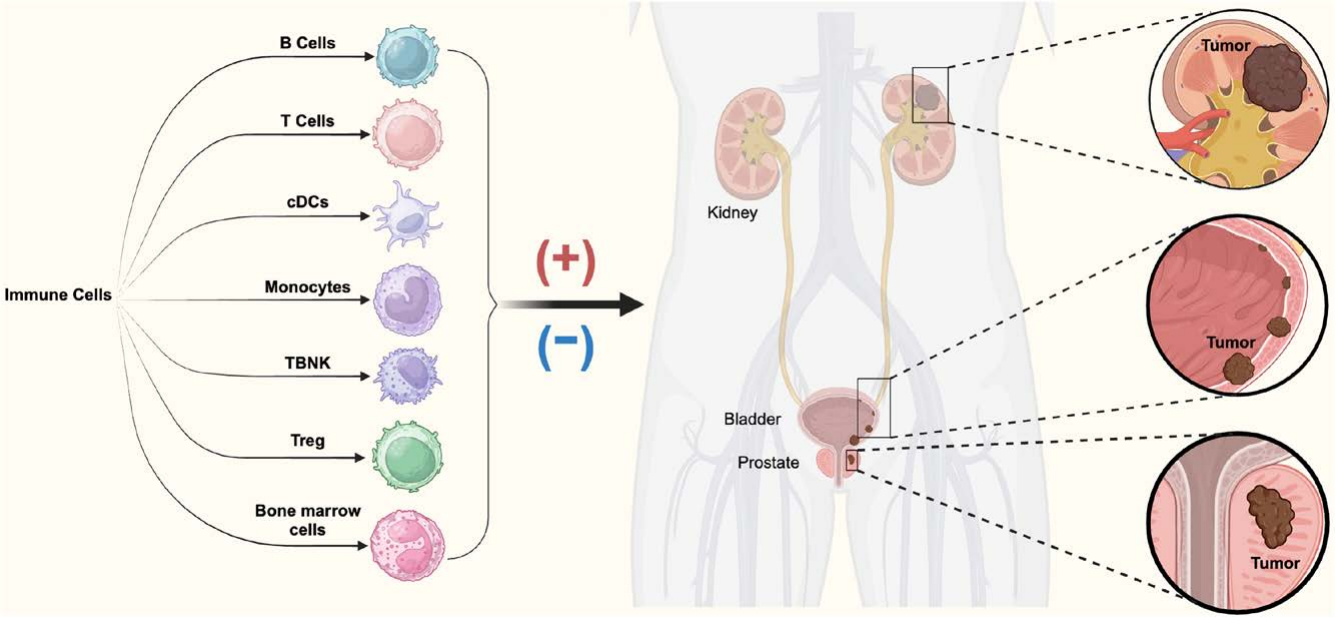
Seven panels of immune cells and the potential causal relationship with 3 types of urogenital system tumors. This figure illustrates the potential causal relationships between 7 categories of immune cells and 3 urogenital system tumors (kidney cancer, bladder cancer, and prostate cancer). Overview of immune cell types and their roles in tumor development. Categories include B cells, T cells, cDCs, monocytes, TBNK, Treg, and bone marrow cells. cDCs: This subset represents conventional dendritic cells with demonstrated associations with tumor immunity. TBNK cells: A combination of T cells, B cells, and natural killer cells contributing to immune surveillance and tumor dynamics. Treg: Regulatory T cells known for suppressing immune responses and potentially facilitating tumor progression. Positive (+) and negative (−) symbols indicate immune cell phenotypes potentially increasing or reducing tumor risks, respectively. The figure was created using BioRender.com by the authors. cDCs = conventional dendritic cells, TBNK = T cells, B cells, and natural killer cells, Treg = regulatory T cells.

Uncontrolled proliferation and spread of abnormal cells are what makes tumor, an extremely complex disease.^[1,2]^ According to epidemiological data, human cancer and the immune system have a complex and close interaction.^[3,4]^ Immunotherapy is another promising method for treating tumors after chemotherapy,^[5]^ including but not limited to blocking so called immune checkpoint blockades ^[6]^ and targeting the tumor immune microenvironment ^[7]^ through immune checkpoints. Immune cells generally contribute positively to tumor resistance, but occasionally they can encourage tumor growth and development.^[8,9]^ Immunotherapy is used to treat cancer by altering the tumor immune microenvironment. The focus of these treatments is on different phenotypes or categories of immune cells within the immune system. The number and composition of immune cells have been shown to be related to specific cancer risks, as shown by research.^[10]^ Immune cells are crucial in the development of kidney cancer, bladder cancer, and PC^[11–15]^ (Fig. 1). Studying the relationship between immune cells and these 3 types of urinary system cancers helps to understand the causes of diseases and corresponding treatment methods, thereby reducing the burden on patients and society. There is currently a lack of research on the link between immune cells and urinary system tumors, and some research findings may be influenced by confounding factors and reverse causal relationships. Hence, it is imperative to delve deeper into the correlation between the immune cells and tumors affecting the urinary system.

Mendelian randomization (MR) analysis is a method based on Mendel’s law of independent assortment that uses genetic variations, such as single nucleotide polymorphisms (SNPs), as instrumental variables (IVs).^[16,17]^ By randomly assigning the alleles of parents to offspring during meiosis, this method can minimize the possibility of confusion and reverse causality.^[18]^ We used immune cell phenotypes as exposure factors and employed the MR analysis to assess the causal association between human immune cells and common urinary system tumors.

## 2. Materials and methods

### 2.1. Study design

**Figure 2.**
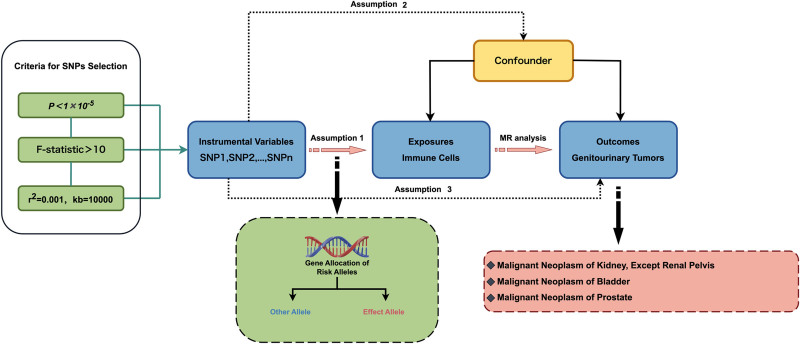
Study design of Mendelian randomization study on the causal relationship between immune cells and genitourinary tumors. This flow diagram outlines the principles of Mendelian randomization (MR) and how genetic variants (SNPs) were used as instrumental variables (IVs) to estimate the causal effect of immune cell phenotypes on the risks of kidney, bladder, and prostate cancers. SNP selection criteria: Variants met thresholds of *P* < 1 × 10^−5^, linkage disequilibrium thresholds (*r*² = 0.001, kb = 10,000), and instrument strength (*F*-statistic > 10). Instrumental variables: Selected SNPs serve as proxies for immune cell exposure to assess their impact on tumor development while controlling for confounders. Outcome analysis: Examines the causal impact of genetically predicted immune cell traits on malignant neoplasms of the kidney (excluding renal pelvis), bladder, and prostate. SNP. *r*² = linkage disequilibrium. This figure was created by the author using the figdraw. IVs = instrumental variables, kb = kilobase, MR = Mendelian randomization, SNPs = single nucleotide polymorphisms.

The study’s design is depicted in Figure 2.

### 2.2. Instrumental variables

Based on the MR analysis of 2 samples, we evaluated the causal relationship between 731 immunophenotypes (7 panels) and 3 types of urinary system tumors (kidney cancer, bladder cancer, and PC). MR analysis employs genetic variation as proxies for risk factors. Hence, for causal inference to be effective, IVs must meet 3 fundamental assumptions: Genetic variation directly links to exposure; (Genetic variation is unrelated to potential confounding factors between exposure and outcomes; and Genetic variation doesn’t influence outcomes through pathways other than exposure.^[19]^

### 2.3. Genetic association of SNPs with 3 types of urinary system tumors risk

Genome-wide association study (GWAS) summary statistics of renal cancer, bladder cancer and PC were from Finnish Biobank data (FinnGen) database. There were 2372 cases and 3,14,193 participants of renal cancer in the FinnGen database. There were 2193 cases and 3,14,193 participants of bladder cancer. There were 15,199 cases and 1,31,266 participants of PC. The 3 types of urinary system tumors were diagnosed and classified according to the International Classification of Diseases for Oncology, 3rd Edition. Cancer cases were identified from the cancer register using this standardized coding system. To address missing data, we used biological annotation software, including the R/Bioconductor package SNPlocs.Hsapiens.dbSNP144.GRCh38, to retrieve SNPs that lacked an rsID (Reference SNP cluster ID) in the original data, and finally obtained 1,98,38,363 SNPs for kidney cancer, 1,98,38,351 SNPs for bladder cancer, and 1,98,23,027 SNPs for PC. The FinnGen database is a vast genomics initiative that examined more than 5,00,000 samples from the Finnish biobank, linking genetic variations with health information to comprehend disease mechanisms and vulnerability. This endeavor entails cooperation among Finnish research institutions, biobanks, and global industry collaborators.^[20]^

### 2.4. Genetic association of SNPs with immune cell phenotype

The summary statistical data for each immune trait in GWAS can be accessed publicly through the GWAS directory, with login numbers ranging from GCST0001391 to GCST0002121.^[21]^ This encompasses a total of 731 immune phenotypes, comprising absolute cell (AC), median fluorescence intensity indicating surface antigen levels, morphological parameters, and relative cell count. The initial immune traits in GWAS were derived from data of 3757 Europeans without overlapping cohorts. Around 22 million genotype SNPs from high-density arrays were incorporated into the Sardinian sequence reference panel and subjected to correlation analysis following adjustment for covariates such as gender, age, and age-2.^[22]^

### 2.5. Statistical analysis

According to recent research,^[22,23]^ the significance level of IVs for each immune trait was set at 1E−05. After removing palindromic and non-SNPs sequences, establish linkage disequilibrium with *r*^2^ < 0.001 and SNPs loci with a genetic distance of 10,000 kb, and select SNPs loci with lower *P*-values. For kidney cancer, bladder cancer and PC, we adjusted the significance level to 5E−08 and applied a similar linkage disequilibrium check (*r*^2^ = 0.001, kb = 10,000). We computed the *F*-statistic for each SNP and excluded those with low *F*-values (<10) to evaluate instrumental strength and minimize potential bias due to ineffective or weak IVs.^[24]^ There were no overlapping individuals between the exposure and outcome studies, as the 2 samples were drawn from distinct populations and datasets: the exposure dataset from Sardinia and the outcome dataset from Finland. Lastly, we excluded SNPs related to potential confounding variables using the PhenoScanner database.

**Figure 3.**
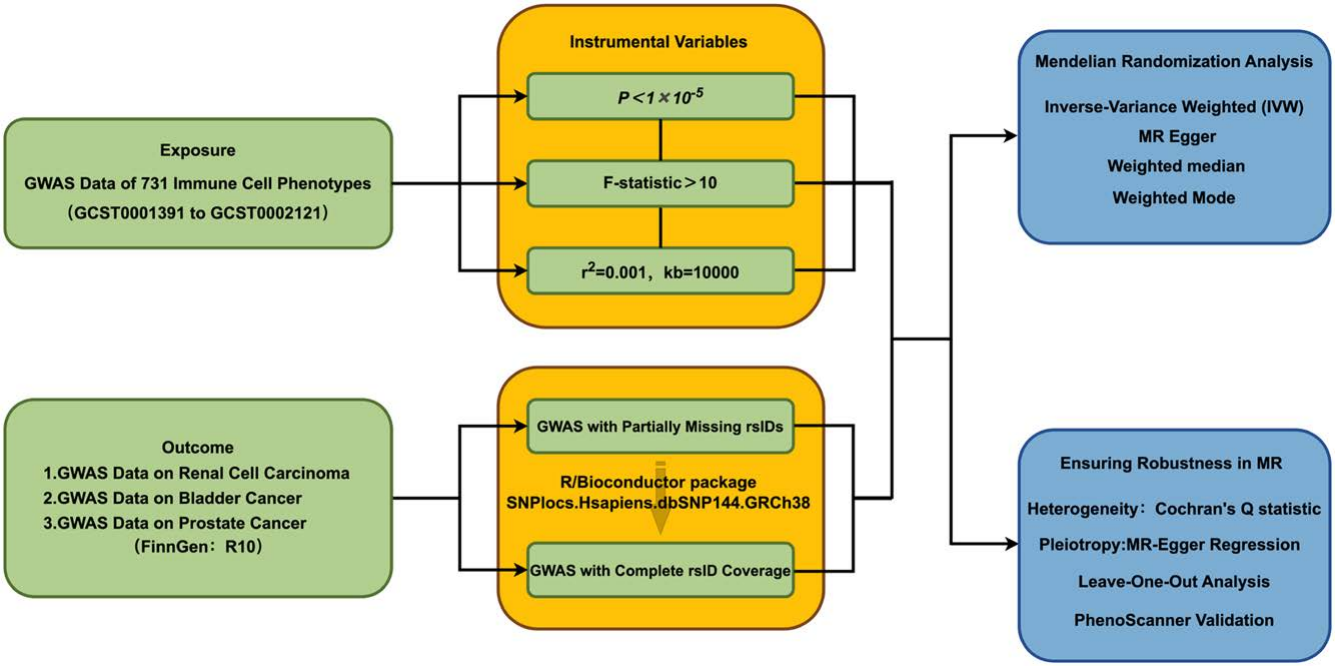
Workflow for identifying immune cell–tumor associations and conducting Mendelian randomization with robustness analyses. The figure provides a detailed explanation of the analysis of causal relationships between immune cell phenotypes and genitourinary tumors, along with robustness assessments. GWAS data extraction: Immune cell phenotypes were obtained from GCST0001391 to GCST0002121. Instrumental variable selection: SNPs with *P* < 1 × 10^−5^, *F*-statistic > 10, and low linkage disequilibrium (*r*² = 0.001, kb = 10,000) were selected. Data harmonization: Missing rsIDs were matched using the R/Bioconductor package SNPlocs.Hsapiens.dbSNP144.GRCh38. Outcome data source: Tumor outcome data were extracted from the FinnGen GWAS database (R10 release). Mendelian randomization: Inverse variance weighted (IVW) analysis was complemented by MR-Egger, weighted median, and weighted mode to assess causality. The figure provides a detailed explanation of the analysis of causal relationships between immune cell phenotypes and genitourinary tumors, along with robustness assessments, including testing heterogeneity using Cochran’s *Q*-statistic, evaluating pleiotropy via the MR-Egger intercept, identifying influential SNPs through leave-one-out analysis, and using PhenoScanner to exclude instrumental variables affected by confounding factors. This figure was created by the author using the figdraw. FinnGen = Finnish Biobank data, GWAS = genome-wide association study, IVW = inverse variance weighted, MR = Mendelian randomization, SNPs = single nucleotide polymorphisms.

To investigate the potential causal link between immune cells and renal, bladder, and PC, we employed various MR analysis techniques. These methods included 4 types: inverse variance weighted (IVW),^[25,26]^ MR-Egger,^[27]^ Weighted median,^[28]^ and Weighted mode.^[29]^ These different techniques can thoroughly investigate the potential causal relationship between immune cells and 3 types of urinary system tumors. Firstly, the IVW method serves as the main method for causal effects in MR studies, demonstrating the robust causal detection and high-quality testing results.^[30]^ Because when the directional pleiotropy of IVs is missing, it provides the most striking estimate.^[31]^ When using the MR-Egger method, the main observation is the intercept term, which is used to measure the level pleiotropy of IVs. When the intercept term approaches zero, the MR-Egger regression model closely resembles the IVW method. Conversely, a significantly nonzero intercept term suggests the presence of horizontal pleiotropy among these IVs.^[32]^ We use false discovery rate (FDR) correction because multiple tests increase the likelihood of Type I errors.^[33]^ Finally, in this study, we interpret the causal relationships using odds ratio (OR). The heterogeneity of IV is estimated using Cochran’s *Q*-statistic and its corresponding *P*-value. Additionally, we implemented a leave-one-out approach by systematically omitting each IVs SNP to detect possible heterogeneous SNPs. Scatter plots were also utilized, assessing the randomness of the experimental design to ensure no noticeable differences or biases between the experimental and control groups. Furthermore, we conducted reverse MR analysis on immune cells identified in the initial MR analysis that displayed causal associations with renal, bladder, and PCs, aiming to assess whether there is a reverse causal relationship between immune cells and the 3 tumors. (Fig. 3) All statistical analyses are conducted using R version 4.3.1.

## 3. Results

### 3.1. Exploration of the causal effect of immune types on kidney cancer, bladder cancer, and prostate cancer

We employed 2-sample MR analysis, with IVW as the primary method and 3 other methods, including MR-Egger, weighted median, and weighted mode, as auxiliary analysis methods, to examine the causal relationship between 731 immunophenotypes and 3 types of urinary system tumors. To further visualize the associations between genetic variants and cancer risks, forest plots were used to display the OR and 95% confidence intervals (CIs) for each identified immunophenotype. Cochran’s *Q* test was used to assess heterogeneity in the data, with results summarized in Tables 1–3. As shown, the *Q* statistics for kidney cancer, bladder cancer, and PC indicate no significant heterogeneity among these results. The specific *Q* values and corresponding *P*-values for each set of SNPs are listed in the tables and at a significance level of .01, 12 immunophenotypes were identified for kidney cancer, 4 for bladder cancer, and 6 for PC. These phenotypes were all adjusted using the FDR method, with *P*_FDR < .05 for all 3 tumors. Additionally, we utilized scatter plots and leave-one-out methodology to respectively showcase variable relationships and isolate variables’ effects (as shown in Figs. S1–S6, Supplemental Digital Content, https://links.lww.com/MD/P910).

**Table 1:** Causal effects of immune cells on renal cancer and sensitivity analysis.

Panel	Exposure	Method	OR (95% CI)	*P*	*P*_FDR_	Heterogeneity	
						*Q*	*Q*-*P*val
B cell	IgD+ CD38−% lymphocyte	Inverse variance weighted	1.101 (1.031–1.177)	.004	.009	22.234	.273
		MR-Egger	1.104 (1.018–1.198)	.028		22.216	.223
		Weighted median	1.102 (1.011–1.201)	.027			
		Weighted mode	1.104 (1.019–1.196)	.025			
	CD20 on IgD− CD38br	Inverse variance weighted	1.067 (1.022–1.114)	.003	.008	11.307	.731
		MR-Egger	1.065 (1.013–1.119)	.027		11.282	.664
		Weighted median	1.060 (0.997–1.128)	.062			
		Weighted mode	1.063 (1.013–1.115)	.026			
	CD25 on B cell	Inverse variance weighted	1.067 (1.017–1.120)	.008	.008	20.588	.663
		MR-Egger	1.052 (0.993–1.114)	.100		19.748	.657
		Weighted median	1.089 (1.009–1.175)	.029			
		Weighted mode	1.073 (1.003–1.148)	.053			
cDC	CD62L− plasmacytoid DC %DC	Inverse variance weighted	0.906 (0.850–0.965)	.002	.007	11.533	.828
		MR-Egger	0.906 (0.830–0.989)	.042		11.532	.775
		Weighted median	0.902 (0.817–0.995)	.040			
		Weighted mode	0.896 (0.821–0.979)	.026			
	CD11c on myeloid DC	Inverse variance weighted	0.904 (0.853–0.959)	7.38E−04	.004	39.435	.094
		MR-Egger	0.908 (0.840–0.981)	.022		39.406	.075
		Weighted median	0.917 (0.842–0.999)	.048			
		Weighted mode	0.931 (0.872–0.995)	.043			
	CD11c on CD62L+ myeloid DC	Inverse variance weighted	0.930 (0.881–0.981)	.008	.009	28.297	.294
		MR-Egger	0.941 (0.877–1.011)	.109		27.990	.260
		Weighted median	0.935 (0.869–1.005)	.069			
		Weighted mode	0.955 (0.896–1.017)	.160			
Maturation stages of T cell	CD4 on TD CD4+	Inverse variance weighted	1.102 (1.027–1.182)	.007	.008	20.166	.632
		MR-Egger	1.118 (0.990–1.262)	.087		20.085	.578
		Weighted median	1.103 (0.985–1.234)	.089			
		Weighted mode	1.115 (0.992–1.254)	.082			
Monocyte	HLA DR on CD14+ CD16+ monocyte	Inverse variance weighted	0.947 (0.916–0.980)	.002	.007	11.897	.853
		MR-Egger	0.960 (0.919–1.004)	.093		11.044	.854
		Weighted median	0.937 (0.891–0.985)	.011			
		Weighted mode	0.955 (0.918–0.994)	.036			
Myeloid cell	HLA DR on CD33dim HLA DR+ CD11b−	Inverse variance weighted	0.924 (0.874–0.977)	.005	.009	12.977	.738
		MR-Egger	0.897 (0.796–1.011)	.095		12.676	.696
		Weighted median	0.916 (0.848–0.990)	.027			
		Weighted mode	0.907 (0.817–1.007)	.086			
TBNK	HLA DR on B cell	Inverse variance weighted	0.929 (0.882–0.979)	.006	.008	12.961	.910
		MR-Egger	0.910 (0.835–0.993)	.046		12.620	.893
		Weighted median	0.931 (0.869–0.998)	.043			
		Weighted mode	0.925 (0.867–0.988)	.030			
Treg	CD127− CD8br AC	Inverse variance weighted	1.205 (1.099–1.320)	6.73E−05	8.07E−04	11.506	.486
		MR-Egger	1.074 (0.898–1.284)	.450		9.355	.589
		Weighted median	1.081 (0.943–1.241)	.264			
		Weighted mode	1.107 (0.964–1.271)	.176			
	CD25 on CD39+ activated Treg	Inverse variance weighted	1.136 (1.037–1.244)	.006	.009	12.313	.341
		MR-Egger	1.127 (0.997–1.274)	.086		12.263	.268
		Weighted median	1.141 (1.011–1.287)	.033			
		Weighted mode	1.144 (1.031–1.270)	.028			

This table presents the Mendelian randomization analysis of the causal effects of immune cell subsets on renal cancer risk. Four statistical methods were applied: Inverse variance weighted (IVW), MR-Egger, weighted median, and weighted mode. For each immune cell exposure, the odds ratios (ORs) and their 95% confidence intervals (95% CI) are reported. *P*-values indicate the statistical significance of the findings, while FDR-adjusted *P*-values (*P*_FDR) account for multiple comparisons. Robustness of the findings was evaluated through heterogeneity and sensitivity analyses. Heterogeneity was assessed using the *Q* statistic and its corresponding *P*-value (*Q*-*P*val) to test consistency across genetic variants. Immune cell exposures analyzed include IgD+ CD38−% lymphocyte, CD20 on IgD− CD38br, and various T cell maturation stages, as well as monocytes and myeloid cells. The causal relationships between these exposures and renal cancer risk were thoroughly examined. Key findings are highlighted with heterogeneity and sensitivity results to ensure robustness.

In this table, “+” denotes positive expression of the marker, while “−” denotes negative expression.

95% CI = 95% confidence interval, IVW = inverse variance weighted, MR = Mendelian randomization, OR = odds ratio, *P* = *P*-value, indicating statistical significance, *P*_FDR = false discovery rate-adjusted *P*-value, *Q* = heterogeneity statistic, *Q*-*P*val = *P*-value for heterogeneity.

**Table 2:** Causal effects of immune cells on bladder cancer and sensitivity analysis.

Panel	Exposure	Method	OR (95% CI)	*P*	*P*_*FDR*_	Heterogeneity	
						*Q*	*Q*-*P*val
B cell	IgD− CD38dim% lymphocyte	Inverse variance weighted	1.081 (1.020–1.145)	.009	.012	15.249	.851
		MR-Egger	1.088 (1.012–1.170)	.034		15.162	.815
		Weighted median	1.077 (0.993–1.169)	.074			
		Weighted mode	1.089 (1.010–1.174)	.036			
	IgD on unsw mem	Inverse variance weighted	0.908 (0.856–0.963)	.001	.006	16.219	.757
		MR-Egger	0.900 (0.827–0.979)	.024		16.122	.709
		Weighted median	0.929 (0.858–1.007)	.074			
		Weighted mode	0.911 (0.843–0.984)	.027			
TBNK	HLA DR+ CD8br AC	Inverse variance weighted	0.940 (0.897–0.985)	.009	.009	32.170	.508
		MR-Egger	0.930 (0.872–0.993)	.037		31.969	.468
		Weighted median	0.928 (0.873–0.987)	.017			
		Weighted mode	0.936 (0.880–0.995)	.041			
cDC	FSC-A on granulocyte	Inverse variance weighted	0.897 (0.836–0.963)	.003	.005	12.733	.889
		MR-Egger	0.883 (0.791–0.985)	.038		12.587	.859
		Weighted median	0.905 (0.816–1.003)	.057			
		Weighted mode	0.898 (0.813–0.992)	.047			

This table presents the causal effects of different immune cell subsets on bladder cancer using Mendelian randomization analysis. The statistical methods used include inverse variance weighted (IVW), MR-Egger, weighted median, and weighted mode. For each immune cell exposure, odds ratios (ORs) with 95% confidence intervals (95% CI) are reported, as well as *P*-values and FDR-adjusted *P*-values (*P*_FDR_) to account for multiple comparisons. Robustness of the findings was evaluated through heterogeneity and sensitivity analyses. Heterogeneity was assessed using the *Q* statistic and its corresponding *P*-value (*Q*-*P*val) to test consistency across instrumental variables. Immune cell subsets analyzed include IgD− CD38dim% lymphocyte, IgD on unswitched memory cells, HLA DR+ CD8br AC, and other dendritic cell (DC) subsets. Key findings are highlighted with heterogeneity and sensitivity results to ensure robustness.

In this table, “+” denotes positive expression of the marker, while “−” denotes negative expression.

95% CI = 95% confidence interval, IVW = inverse variance weighted, MR = Mendelian randomization, OR = odds ratio, *P* = *P*-value, *P*_FDR_ = false discovery rate-adjusted *P*-value, *Q* = heterogeneity statistic, *Q*-*P*val = heterogeneity *P*-value.

**Table 3:** Causal effects of immune cells on prostate cancer and sensitivity analysis.

Panel	Exposure	Method	OR (95% CI)	*P*	*P*_FDR_	Heterogeneity	
						*Q*	*Q*-*P*val
B cell	CD19 on IgD− CD38−	Inverse variance weighted	1.080 (1.033–1.129)	6.21E−04	.002	22.381	.378
		MR-Egger	1.071 (0.986–1.163)	.120		22.320	.323
		Weighted median	1.053 (0.984–1.127)	.132			
		Weighted mode	1.036 (0.951–1.128)	.425			
	CD25 on IgD+ CD38−	Inverse variance weighted	0.974 (0.960–0.988)	2.59E−04	.002	30.199	.305
		MR-Egger	0.971 (0.955–0.987)	.001		29.421	.292
		Weighted median	0.980 (0.960–1.001)	.057			
		Weighted mode	0.975 (0.960–0.990)	.003			
	CD27 on CD20−	Inverse variance weighted	1.040 (1.010–1.072)	.009	.009	9.209	.817
		MR-Egger	1.025 (0.986–1.065)	.232		7.617	.868
		Weighted median	1.037 (0.993–1.082)	.098			
		Weighted mode	1.037 (0.999–1.076)	.076			
cDC	CD86+ plasmacytoid DC %DC	Inverse variance weighted	1.053 (1.013–1.094)	.009	.010	25.425	.439
		MR-Egger	1.100 (1.028–1.177)	.010		23.023	.518
		Weighted median	1.052 (0.996–1.112)	.072			
		Weighted mode	1.056 (0.991–1.125)	.103		29.424	.205
Treg	CD25hi CD45RA+ CD4 not Treg AC	Inverse variance weighted	0.962 (0.937–0.989)	.006	.009	28.963	.182
		MR-Egger	0.955 (0.920–0.992)	.025			
		Weighted median	0.965 (0.929–1.002)	.067			
		Weighted mode	0.971 (0.937–1.006)	.118		11.760	.896
	CD127 on CD28− CD8br	Inverse variance weighted	0.952 (0.920–0.984)	.004	.007	11.760	.859
		MR-Egger	0.951 (0.901–1.005)	.090		22.381	.378
		Weighted median	0.949 (0.904–0.996)	.034		22.320	.323
		Weighted mode	0.950 (0.906–0.995)	.045			

This table summarizes the causal effects of different immune cell subtypes on prostate cancer using Mendelian randomization methods. The 4 methods used are inverse variance weighted (IVW), MR-Egger, weighted median, and weighted mode. For each immune cell exposure, odds ratios (ORs) with 95% confidence intervals (95% CI) are reported, as well as *P*-values and FDR-adjusted *P*-values (*P*_FDR_) to account for multiple comparisons. Robustness of the findings was evaluated through heterogeneity and sensitivity analyses. Heterogeneity was assessed using the *Q* statistic and its corresponding *P*-value (*Q*-*P*val) to test consistency across genetic variants. The immune cell exposures analyzed include CD19 on IgD− CD38−, CD25 on IgD+ CD38−, CD27 on CD20−, and others. Key findings are highlighted with heterogeneity and sensitivity results to ensure robustness.

In this table, “+” denotes positive expression of the marker, while “−” denotes negative expression.

95% CI = 95% confidence interval, IVW = inverse variance weighted, MR = Mendelian randomization, OR = odds ratio, *P* = *P*-value, *P*_FDR_ = false discovery rate-adjusted *P*-value, *Q* = heterogeneity statistic, *Q*-*P*val = heterogeneity *P*-value.

### 3.2. The causal relationship between immune cell phenotype and kidney cancer

Under the conditions of IVW method, immunophenotypes with a significance of .01 were screened, and we detected 12 immunophenotypes associated with kidney cancer. After IVW treatment, the *P*-values of these immunophenotypes were all <.05 after FDR correction, with 3 in the B cell panel, 3 in the conventional dendritic cell (cDC) panel, 1 in the mature stages of T cell panel, 1 in the monocyte panel, 1 in the myeloid cell panel, 1 in the TBNK panel, and 2 in the regulatory T cell (Treg) panel.

**Figure 4.**
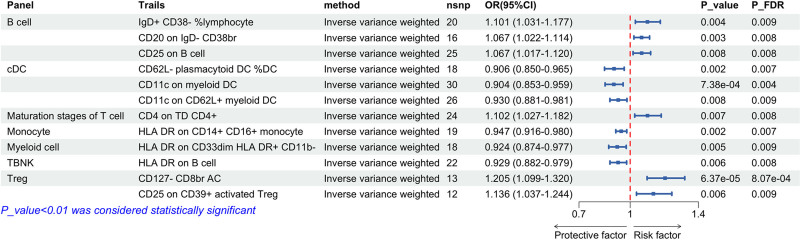
Forest plot using the IVW method illustrate a causal relationship between renal cancer and immune traits. This forest plot analyzing the association between immune-cell phenotypes and renal cancer risk using the inverse variance weighted (IVW) method. The plot displays the odds ratios and 95% confidence intervals for each immune trait, with significance indicated by probability values and *P*-values adjusted by the false discovery rate. Each row indicates a specific phenotype, with point estimates represented by squares and corresponding CIs by horizontal lines. This figure highlights both protective factors (OR < 1) and risk factors (OR > 1) for renal cancer. Statistically significant results (*P* < .01) are highlighted, with details explained at the bottom of the table. CD8br = CD8 bright, cDC = conventional dendritic cells, CIs = confidence intervals, IVW = inverse variance weighted, OR = odds ratio, *P*-value = probability value, *P*_FDR = *P*-value adjusted by the false discovery rate, TBNK = T cells, B cells, and natural killer cells, Treg = regulatory T cells.

The results from the aforementioned 4 MR analysis methods indicated that 6 immunophenotypes were significantly associated with an increased risk of kidney cancer (Fig. 4). Using the IVW method, it was found that the CD127 CD8br AC in Treg (OR = 1.205, 95% CI: 1.099–1.320, *P* = 6.73 × 10^−5^); CD25 on CD39+ activated Treg (OR = 1.136, 95% CI: 1.037–1.244, *P* = .006). In the mature stages of T cell panel, the IVW method measured CD4 on TD CD4+ (OR = 1.102, 95% CI: 1.027–1.182, *P* = .007). In the B cell panel, immunoglobulin D (IgD)+ CD38−% lymphocyte was measured using the IVW method (OR = 1.101, 95% CI: 1.031–1.177, *P* = .004). CD20 on IgD CD38br (OR = 1.067, 95% CI: 1.017–1.120, *P* = .008), CD25 on B cell (OR = 1.067, 95% CI: 1.022–1.114, *P* = .004). There is a significant correlation with the increased risk of kidney cancer. We found that 6 immunophenotypes were also found to reduce the risk of RCC. In the TBNK panel, HLA DR on B cell was obtained using IVW method (OR = 0.929, 95% CI: 0.882–0.979, *P* = .006). In the myeloid cell panel, the IVW method was used to obtain HLA DR on CD33dim HLA DR+ CD11b− (OR = 0.924, 95% CI: 0.874–0.977, *P* = .005). In the monocyte panel, the HLA DR on CD14+ CD16+ monocyte calculated using the IVW method (OR = 0.947, 95% CI: 0.916–0.980, *P* = .002). In the cDC panel, CD62L plasmacytoid DC% DC was calculated using the IVW method (OR = 0.906, 95% CI: 0.850–0.965, *P* = .002). CD11c on myoid DC (OR = 0.904, 95% CI: 0.853–0.959, *P* = 7.38 × 10^−4^). Using the IVW method, CD11c on CD62L+ myoid DC was obtained (OR = 0.930, 95% CI: 0.881–0.981, *P* = .008; Table 1).

### 3.3. The causal relationship between immune cell phenotype and bladder cancer

**Figure 5.**
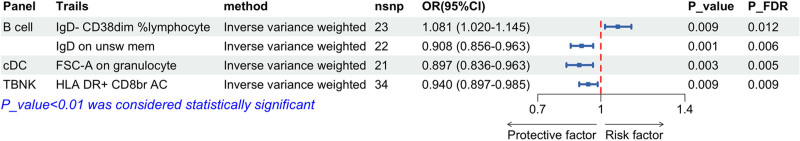
Forest plot using the IVW method illustrate a causal relationship between bladder cancer and immune traits. This forest plot analyzing the association between immune-cell phenotypes and bladder cancer risk using the inverse variance weighted (IVW) method. The plot displays the odds ratios and 95% confidence intervals for each immune trait, with significance indicated by probability values and *P*-values adjusted by the false discovery rate. Each row indicates a specific phenotype, with point estimates represented by squares and corresponding CIs by horizontal lines. This figure highlights both protective factors (OR < 1) and risk factors (OR > 1) for renal cancer. Statistically significant results (*P* < .01) are highlighted, with details explained at the bottom of the table. AC = activated cells, CD38dim = CD38 diminished, CI = confidence interval, FSC-A = forward Scatter-area, HLA = human leukocyte antigen, OR = odds ratio, *P*-value = probability value, *P*_FDR = *P*-value adjusted by the false discovery rate, unsw mem = unswitched memory.

Under the same significant conditions and after FDR correction, there were 4 immunophenotypes associated with bladder cancer (Fig 5). In the B cell panel, IVW measured IgD− CD38dim% lymphocyte (OR = 1.081, 95% CI: 1.020–1.145, *P* = .009), which was significantly associated with the increased risk of bladder cancer. There are 3 immunophenotypes that are closely related to the reduced risk of bladder cancer. In the TBNK panel, HLA DR+ CD8br AC measured by IVW method (OR = 0.940, 95% CI: 0.897–0.985, *P* = .009). In the B cell panel, IgD on unsaw mem measured by IVW method (OR = 0.908, 95% CI: 0.856–0.963, *P* = .001). In the cDC panel, the FSC-A on granulocyte measured by IVW method (OR = 0.897, 95% CI: 0.836–0.963, *P* = .003; Table 2).

### 3.4. The causal relationship between immune cell phenotype and prostate cancer

**Figure 6.**
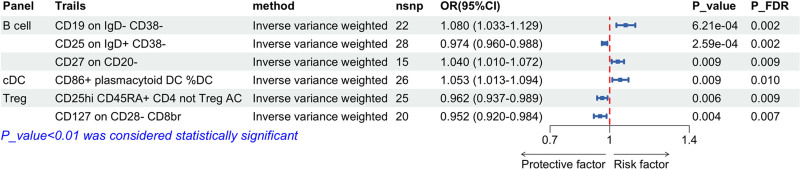
Forest plot using the IVW method illustrate a causal relationship between prostate cancer and immune traits. This forest plot analyzing the association between immune-cell phenotypes and prostate cancer risk using the inverse variance weighted (IVW) method. The plot displays the odds ratios and 95% confidence intervals for each immune trait, with significance indicated by probability values and *P*-values adjusted by the false discovery rate. Each row indicates a specific phenotype, with point estimates represented by squares and corresponding CIs by horizontal lines. CD86+ plasmacytoid DC %DC: CD86 positive plasmacytoid dendritic cells as a percentage of total dendritic cells. CD25hi CD45RA+ CD4 not Treg AC: CD4 positive cells with high CD25 expression and positive for CD45 Regulatory Isoform A, which are not activated regulatory T cells. This figure highlights both protective factors (OR < 1) and risk factors (OR > 1) for renal cancer. Statistically significant results (*P* < .01) are highlighted, with details explained at the bottom of the table. CI = confidence interval, IVW = inverse variance weighted, OR = odds ratio, *P*-value = probability value, *P*_FDR = *P*-value adjusted by the false discovery rate, Treg = regulatory T cells.

Under the same conditions as the previous 2, we found 6 immunophenotypes associated with prostate development (Fig 6), among which 3 immunophenotypes were risk factors for PC. In the B cell panel, there were 2 types, and CD19 on IgD CD38− (OR = 1.080, 95% CI: 1.033–1.129, *P* = 6.21 × 10^−4^) and CD27 on CD20− (OR = 1.040, 95% CI: 1.010–1.072, *P* = .009) were obtained using IVW. In the cDC panel, the CD86+ plasmacytoid DC% DC obtained by the IVW method (OR = 1.053, 95% CI: 1.013–1.094, *P* = .009). The other 3 are protective factors for PC incidence: in the B cell panel, CD25 on IgD+ CD38− (OR = 0.974, 95% CI: 0.960–0.988, *P* = 2.59 × 10^−4^) measured by IVW method. In the Treg panel, 1 immune cell phenotype was identified as CD25hi CD45RA+ CD4 not Treg AC using the IVW method (OR = 0.962, 95% CI: 0.937–0.989, *P* = .006), while the other was CD127 on CD28− CD8br (OR = 0.952, 95% CI: 0.920–0.984, *P* = .004; Table 3).

### 3.5. Causal relationships 3 common urinary system tumors on immunophenotypes

We employed the same 4 MR methods as in the forward analysis to perform reverse causal analysis on the association between immunophenotypes and 3 common urinary system tumors. Under the condition of *P* < .01, there was no causal relationship between kidney cancer, bladder cancer, and PC as exposure factors and immunophenotypes.

### 3.6. The causal relationship between immune cell phenotypes and 3 common urinary system tumors: a summary of key findings

In this study, we performed comprehensive 2-sample MR analyses to elucidate potential causal relationships between immune cell phenotypes and the risk of kidney, bladder, and PC. Our primary IVW analysis, supported by MR-Egger, weighted median, and weighted mode methods, yielded robust and consistent findings. For kidney cancer, 12 immunophenotypes reached statistical significance (*P* < .01), with 6 associated with increased risk and 6 presenting a protective effect. Notably, risk-enhancing phenotypes included CD127 CD8br AC in Treg and IgD+ CD38−% lymphocyte on B cells, while protective phenotypes involved markers such as HLA-DR on myeloid and monocyte subsets. Similarly, for bladder cancer, 4 immunophenotypes were implicated, with IgD− CD38dim% lymphocyte on B cells indicating increased risk and HLA-DR+ CD8br AC and IgD expression on unswitched memory B cells exhibiting protective effects. In PC, 6 significant immunophenotypes emerged, with 3 elevating risk (e.g., CD19 on IgD− CD38− on B cells) and 3 reducing it (e.g., CD25 on IgD+ CD38− on B cells). Heterogeneity tests showed no substantial variations in SNP effects across these analyses, and our reverse MR analyses did not reveal any reverse causal effects of these malignancies on immunophenotypes. These findings highlight specific immune markers that may contribute to oncogenesis or confer protection, providing valuable insights for future immunological and clinical research.

## 4. Discussion

This research utilized abundant publicly available genetic data to explore the correlation between 731 immune cell types and 3 prevalent urinary system malignancies. It represents the inaugural bidirectional MR analysis investigating the connection between multiple immune traits and common malignant tumors of the urinary system. Under significance criteria of <.01, and following FDR correction (*P*_FDR < .05), we identified 12, 4 and 6 immunophenotypes associated causally with renal cancer, bladder cancer, and anterior adenocarcinoma, respectively.

Our research found that increased expression of IgD+ CD38−% lymphocyte, CD20 on IgD− CD38br, and CD25 on B cell within the B-cell subset was associated with an elevated risk of renal malignancy. Based on our understanding, these phenotypes respectively correspond to Naive B cells, Plasmablasts, and Activated B cells, representing different differentiation stages or states of B cells. Overall, some studies indicate that B cells play a role in renal cell carcinoma (RCC), which features a complex tumor immune microenvironment; however, the mechanism remains incompletely understood.^[11]^ Part of the literature supports our conclusions, showing that B cells can inhibit anti-tumor immunity,^[34]^ and certain researchers further assert that, unlike other malignancies, high expression of B-cell immunophenotypes may lead to poorer survival in RCC.^[35]^ In addition, some studies report that interleukin‐10 (IL-10) – secreting B cells are crucial in RCC pathogenesis and that IL-10 can suppress anti-tumor immunity, thereby promoting immune escape.^[36,37]^ The phenotype characteristics of IL-10+ B cells in the microenvironment of tumor infiltration exhibit low expression of CD38 and IgD,^[38]^ aligning with our findings and further underscoring the key role of certain B-cell differentiation stages or states as risk factors in RCC progression. From the cellular perspective, these 3 B-cell stages can influence renal cancer risk via different pathways, consistent with related research. For instance, plasmablasts were shown to possibly suppress T-cell activity by secreting immunosuppressive cytokines (e.g., IL-10), which may indirectly facilitate tumor progression.^[39]^ Even though some existing work suggests a dual role of B cells in RCC,^[40]^ our new findings support 1 side of the scholarly debate. Meanwhile, we observed that CD127− CD8br AC and CD25 on CD39+ activated Tregs, and CD4 on TD CD4 in the T-cell maturation panel were correlated with an increased risk of renal carcinoma. Some scholars have pointed out that the frequency of Treg is higher in peripheral blood mononuclear cells of renal cancer patients is higher,^[41,42]^ partially supporting our viewpoint. In Tregs, CD25 is expressed both peripherally and within RCC tumors, with higher levels in the tumor microenvironment. In RCC, Tregs often co-express CD4 and CD25.^[43]^ Other research suggests that CD4+ CD25+ suppressive T cells (TS cells) can inhibit the immune response against antigens, thus maintaining immune homeostasis.^[44]^ We cannot help but question whether this maintenance of homeostasis might contribute to RCC onset. Consequently, the CD4 on TD CD4 in the T-cell panel’s maturation stage can reasonably be interpreted as a risk factor for RCC. Nonetheless, contradictory findings exist. For instance, the phenotype CD127− CD8br AC may represent a specialized subgroup of CD8+ Treg cells that exert potent cytotoxic effects against tumor cells,^[45]^ a discrepancy possibly resulting from different patient cohorts or experimental protocols. In contrast, we identified 6 immunophenotypes as protective factors against RCC. Within the cDC panel, there were 3 phenotypes: CD62L plasmacytoid DC% DC, CD11c on myoid DC, and CD11c on CD62L+ myoid DC. Jan P. Böttcher et al noted that cDCs play a crucial role in recognizing and capturing tumor-associated antigens and presenting them to T cells. This antigen presentation process is pivotal in tumor immunosurveillance and anti-tumor immune responses, helping to activate and modulate T cells and thus combat tumor growth and spread.^[46]^ From the standpoint of overall cell types, this aligns with our perspective. Among these cDC phenotypes, CD11c merits particular attention; studies suggest CD11c can serve as a marker for dendritic cells (especially conventional DCs) and macrophages, and its expression and function have drawn significant interest in immune-related disease, infection, and tumor immunity research.^[47]^ Another phenotype, CD62L of cDCs, s part of the l-selectin family and is essential for regulating cell migration and homing, particularly for lymphocytes in peripheral blood circulation and movement between lymphoid organs.^[48]^ However, research directly probing CD62L’s specific role in classical dendritic cells remains limited, highlighting the need for further experiments and observations, which could serve as a future direction for RCC research. In addition, our findings identify HLA DR on B cell, HLA DR on CD33dim, HLA DR+ CD11b−, and HLA DR on CD14+ CD16+ monocyte as protective phenotypes in RCC. It is worth noting that although they belong to different cell panels, the common HLA DR phenotype deserves our high attention. HLA DR molecules are expressed on tumor cells of various types of cancer and are associated with good tumor prognosis.^[49]^ The study by scholars such as Lichen Teng suggests that RCC cell culture medium can inhibit the maturation of DCs and impair their function,^[50]^ indirectly suggesting that HLA DR is protective in RCC; both lines of evidence support our conclusions from distinct angles. Nevertheless, gaps persist in HLA-DR–focused RCC studies, necessitating further scholarly exploration. However, there are still certain shortcomings in the exploration of HLA-DR and renal cancer research, and further exploration is needed. Of note, regarding HLA DR on CD14+ CD16+ monocyte, we know monocytes subdivide into classical (CD14+ CD16−), nonclassical (CD14− CD16+), and intermediate (CD14+ CD16+) subsets.^[21]^ Although intermediate monocytes account for only 10% of pro-inflammatory monocytes, studies have shown that CD14+ CD16+ DR++ monocytes are the main producers of tumor necrosis factor in human blood.^[51]^ Tumor necrosis factor is a cytokine secreted by multiple cell types, including macrophages, T cells, B cells, and natural killer cells originally identified capable of inducing tumor cell necrosis.^[52,53]^ This further corroborates our conclusion that HLA DR on CD14+ CD16+ monocyte is a protective factor against renal cancer. For the other 2 HLA-DR–associated protective factors, 2 separate studies indirectly validate our findings: 1 shows that HLA-DR–low or–negative CD33dim/CD11b− cells are generally considered myeloid-derived suppressor cells that release immunosuppressive agents to inhibit effector T cells, facilitating tumor immune evasion^[54]^; another team discovered an increased proportion of myeloid-derived suppressor cells in RCC patients correlates with unfavorable prognosis.^[55]^ However, the function of HLA-DR on B cells in RCC remains unexamined in dedicated studies.

Regarding the relationship between immune cells and bladder cancer, we found that higher levels of IgD− CD38dim% lymphocyte in the B-cell panel, correlated with an increased risk of bladder carcinoma. Compared with the protective immunophenotype IgD on unsw mem (unswitched memory B cells) identified in this study, the IgD− CD38dim% lymphocyte subset is IgD negative. IgD is believed to be involved in the activation of B cells, and secreted IgD is rarely found in serum, indicating that IgD may mainly act as a signaling module to control the activation of B cells for antigen binding.^[56]^ We know that CD38 is a transmembrane glycoprotein abundant on immune cell surfaces, especially on plasma cells and plasmablasts. Ali Zirakzadeh et al found that an increased number of tumor-associated CD38 + cells in bladder cancer pathology may exert a positive impact on survival in urothelial bladder carcinoma patients,^[57]^ which to some extent is consistent with our research results. So, the theory that egged on “unsaw mem,” also known as “unswitched memory B cells expressing IgD,” is a protective factor for bladder cancer also has some rationality. Two other protective factors for bladder cancer are FSC-A on granulocyte in the cDC panel, and HLA DR+ CD8br AC (absolute count) in the TBNK (T-cell, B-cell, NK cell) panel. FSC-A (forward scatter amplitude) measures cell scattering in response to a laser beam; under certain conditions, a higher scattering parameter indicates larger or more complex cDC morphology. Based on the aforementioned inhibitory effect of dendritic cells in tumors,^[46]^ we may speculate that larger or more complex cDCs could confer greater anti-tumor activity. As for the latter, previous discussions in RCC noted that elevated HLA-DR+ expression is related to favorable outcomes in malignancies.^[49]^ Additionally, Vestein Thorsson et al have indicated that in many tumors, new antigen loads correlate with lower levels of Tregs, mast cells, dendritic cells, and memory B cells,^[58]^ consistent with our data. HLA-DR+ CD8+ T cells generally represent a highly activated state with enhanced cytotoxic function, and flow cytometry often designates CD8br plus HLA-DR+ as a hallmark of “tumor-reactive” T cells. In solid tumors, including bladder cancer, a higher infiltration of HLA-DR+ CD8+ T cells is linked to more robust anti-tumor immunity and better clinical prognosis.^[59]^ Moreover, a higher fraction of activated CD8+ T cells in the bladder tumor microenvironment correlates with improved response to checkpoint inhibitors (e.g., PD-1/PD-L1 blockers) and more favorable clinical outcomes.^[59,60]^ Although dedicated studies on these 4 specific phenotypes in bladder cancer remain limited, we can hypothesize that further refinement of B-cell subset characterization may offer deeper insight into humoral immunity and antibody-mediated pathways within the bladder tumor environment. Cooperative interactions between activated CD8+ T cells and highly functional cDCs are crucial in enhancing anti-tumor immunity and improving responses to immunotherapeutics.

Our research also yielded some results regarding the role of immune cell phenotype in PC. The increase in CD19 on IgD− CD38− and CD27 on CD20− immune phenotypes in the B cell panel leads to an increased risk of PC. In the B cell panel, we have learned that IL-10 can be produced, which has the effect of reducing immune suppression against tumors and enhancing immune escape.^[36,37]^ Based on this, it has been reported that IL-10 expression and the frequency of CD19+ IL-10+ B cells are higher in PC tissues than in benign prostatic hyperplasia, providing factual grounds for our pathological assessment of PC risk.^[61]^ This will provide us with a certain factual basis for determining the risk factors of PC at the pathological level. However, dedicated analyses of the “CD19 on IgD− CD38−” or “CD25 on IgD+ CD38−” subsets are sparse. Existing literature indicates that tumor-associated B cells (TAB) in the prostate tumor microenvironment may either foster inflammation and tumor progression or contribute to anti-tumor responses via antigen presentation and T-cell assistance, depending on their activation status and local context.^[62]^ Our current findings regarding these specific phenotypes help refine the understanding of B-cell involvement in PC. Regarding our view that the CD86+ plasmacytoid DC% DC immune phenotype is a risk factor for PC, some studies have pointed out that in the DC of PC, DC is an important participant in the dysfunction of its immunosuppressive microenvironment.^[63–68]^ In a randomized controlled trial administering different dosages of recombinant human granulocyte/macrophage colony-stimulating factor (rhGM-CSF) to patients with biochemically relapsed PC, those in group B (receiving 250 µg/m^2^ thrice weekly) had reduced plasmacytoid DC counts,^[69]^ partially explaining our identified risk factor. Among the protective factors we identified for PC, CD25hi CD45RA+ CD4 not Treg AC deserves attention. While the phenotypes of Tregs remain a topic of debate, CD4+ CD25hi Tregs are widely recognized for their potent immunosuppressive activity and their role in fostering tumor progression through inhibition of anti-tumor immune responses, potentially conflicting with the conclusions drawn in our study. However, per prevailing consensus, the presence of CD45RA+ may denote a resting or unstimulated subset. Some reports note that immature T cells exhibit CCR7+ CD45RA+ on their surface,^[70,71]^ Consequently, we propose that CD4+ CD25^hi Tregs with CD45RA+ on the surface might act as a protective factor in PC, though further experiments are needed to confirm our hypothesis. CD127 (also known as the IL-7 receptor α chain) is a cell-surface molecule in the leukocyte common product family. Regarding CD127 on CD28− CD8br Tregs, it is highly expressed on CD8+ T cells but not prominently on CD28-negative cells. Its interplay with CD28 is predominantly regulated. Notably, in numerous solid tumors, a high infiltration of activated CD8⁺ tumor-infiltrating lymphocytes often correlates with better outcomes due to their cytotoxic capacity.^[72,73]^ This observation partly aligns with our findings, yet the complex phenotype of CD127 on CD28− CD8br Tregs warrants further investigation.

Advances in global healthcare are driving steady progress in the diagnostic and staging of malignancies, particularly urological tumors. Key modalities include tissue biopsy, pathological analysis, and advanced imaging techniques. Taking the European Association of Urology 2024 guidelines on renal, bladder, and PC as an example^[74–76]^: for renal tumors, the R.E.N.A.L. scoring system is recommended to evaluate anatomical complexity^[74]^; for bladder cancer, the VI-RADS system based on multiparametric MRI (mpMRI) provides a standardized assessment of muscle invasion and tumor grading^[77]^; and in PC diagnosis, both Gleason scoring and the Prostate Imaging Reporting and Data System (PI-RADS) have become routine tools for tumor risk stratification.^[76]^ Nevertheless, immunodiagnostics and immunotherapy have yet to attain mainstream status; although the diagnostic and assessment methods recommended in these guidelines are widely accepted, they still present specific applicability limitations. Our identification of 22 immune cell phenotypes with causal links to urological malignancies provides a foundation for future applications. These phenotypes could be incorporated into independent diagnostic frameworks or integrated with established imaging techniques, such as CT or MRI, to improve comprehensive malignancy evaluation. Factoring in immunological parameters may offer clinicians a more holistic perspective on malignant diseases, potentially guiding future research toward new, expanded directions.

## 5. Conclusion

Our study is the first to employ a MR approach to investigate correlations between immune cell phenotypes and 3 common urinary system malignancies. Through rigorous tests for horizontal pleiotropy and heterogeneity, we minimized confounding factors and reverse causality in our findings. Moreover, the study highlights specific immune cell phenotypes that may serve as biomarkers for identifying high-risk patient subgroups, holding significant implications for clinical decision-making and personalized therapeutic strategies. At the same time, the results underscore the need for further validation in larger and more diverse patient populations to ensure robustness and generalizability.

Nonetheless, this research has several limitations. Despite our inclusion of 731 immunophenotypes, certain findings remained incomplete due to data constraints. Additionally, the lack of stratification by sex and age in the tumor datasets may affect the accuracy and broader applicability of our conclusions. Our GWAS data, primarily derived from Sardinian and Finnish populations, constrains the generalizability of these results to other groups; however, the findings may still offer insights for other regions within Eurasia that share similar genetic or environmental factors. Another notable shortcoming is the absence of detailed information on varying exposure periods and levels of immune cell phenotypes, making it unclear whether similar associations would persist under different conditions. We plan to validate our results in larger, more diverse patient cohorts to further examine their robustness and applicability. Moreover, our criterion of *P* < 1 × 10^−5^ for selecting IVs in immune cell phenotypes may not be sufficiently stringent, emphasizing the need for future research with greater sample sizes and more comprehensive MR analyses to deepen our understanding of the relationships between immune cell phenotypes and risks of renal, bladder, and PCs.

Looking ahead, our research will likely focus on several key directions. First, we aim to leverage additional publicly available GWAS datasets, encompassing urinary system malignancies as well as other tumors and human immune cell phenotypes, to explore regional differences in immune cell phenotypes. Second, we plan to build on this study by experimentally validating immune phenotypes that have shown causal relationships with common urinary system malignancies, comparing their expression profiles between benign diseases and malignant tumors within the same organ. If sufficiently powered analyses reveal statistically significant differences, these findings could provide new avenues for the diagnosis and prognostic evaluation of urinary system tumors. Finally, we will investigate how these immune phenotype expression differences can be integrated with current prognostic scoring systems or diagnostic models, thereby facilitating earlier tumor detection and assessment. Collectively, these directions warrant further in-depth exploration to offer more robust, evidence-based guidance for clinical decision-making and personalized healthcare.

## Author contributions

**Conceptualization:** Jingjing Tu, Jiaye Long, Lijun Guo.

**Data curation:** Zhaohui Liu.

**Formal analysis:** Zhaohui Liu.

**Funding acquisition:** Jingjing Tu, Jiaye Long.

**Investigation:** Zhaohui Liu.

**Methodology:** Jingjing Tu, Jiaye Long, Lijun Guo.

**Project administration:** Jingjing Tu, Jiaye Long, Lijun Guo.

**Resources:** Jingjing Tu, Jiaye Long, Lijun Guo.

**Software:** Jingjing Tu, Jiaye Long, Lijun Guo.

**Supervision:** Jingjing Tu, Jiaye Long, Lijun Guo.

**Validation:** Jingjing Tu, Jiaye Long, Lijun Guo.

**Visualization:** Jingjing Tu, Jiaye Long, Lijun Guo.

**Writing** – **original draft:** Zhaohui Liu.

**Writing** – **review & editing:** Jiaye Long, Lijun Guo.

## Supplementary Material


