# Supplementary material for: Causal relationships between immune cells and common urinary system tumors: A bidirectional analysis of Mendelian randomization
Source: Medicine (Baltimore). 2025 Sep 12;104(37):e44297. doi: 10.1097/MD.0000000000044297 (PMC12440413; doi:10.1097/MD.0000000000044297)
Supplement: Supplementary file 1 [file medi-104-e44297-s001.pdf]

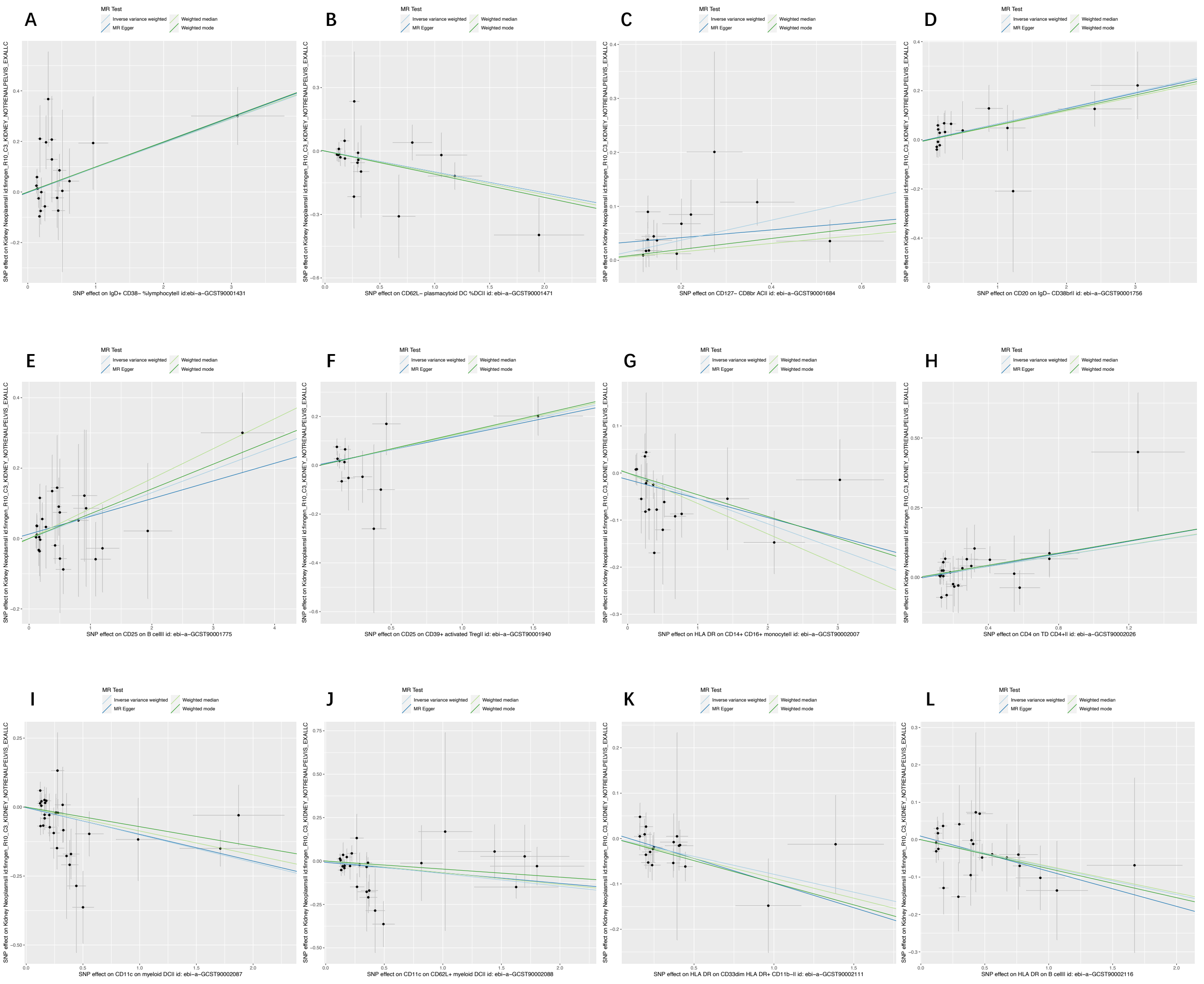

S1 Fig. Scatter plots of immune cell markers in renal cancer This figure presents scatter plots illustrating the association between immune cell markers and renal cancer. Each scatter plot shows the SNP effect sizes on the immune cell markers and their association with kidney cancer risk, analyzed using **Inverse variance weighted (IVW)**, **MR Egger**, **weighted median**, and **weighted mode** methods. **Panels A to L**: The x-axis represents SNP effect sizes on immune cell markers, while the y-axis represents SNP effect sizes on kidney cancer risk (**log(OR)**). Each dot represents an individual SNP. The slope of the regression line indicates the causal effect estimate. Positive slopes suggest risk factors (**OR > 1**), while negative slopes suggest protective factors (**OR < 1**). (A) IgD+ CD38− %lymphocyte on B cell on renal cancer (B) CD62L− plasmacytoid Dendritic Cells on renal cancer (C) CD127− CD8br absolute count on renal cancer (D) CD20 on IgD− CD38br on B cell on renal cancer (E) CD25 on B cell on renal cancer (F) CD25 on CD39+ activated Treg on renal cancer (G) HLA DR on CD14+ CD16+ monocyte on renal cancer (H) CD4 on TD (terminally differentiated) CD4+ T cell on renal cancer (I) CD11c on myeloid DC (Dendritic Cell) on renal cancer (J) CD11c on CD62L+ myeloid dendritic cell on renal cancer (K) HLA DR on CD33dim HLA DR+ CD11b− Myeloid cell on renal cancer (L) HLA DR on B cell on renal cancer

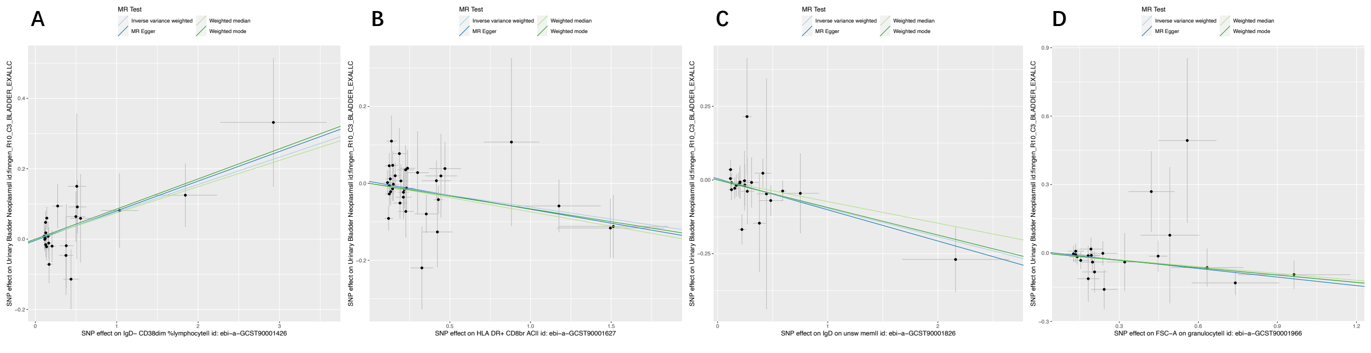

S2 Fig. Scatter plots of immune cell markers in bladder cancer This figure displays scatter plots showing the relationship between immune cell markers and bladder cancer. SNP effect sizes on immune markers and their contribution to bladder cancer risk were analyzed using **Inverse variance weighted (IVW)**, **MR Egger**, **weighted median**, and **weighted mode** methods. **Panels A to D**: The x-axis represents SNP effect sizes on immune cell markers, while the y-axis represents SNP effect sizes on bladder cancer risk (**log(OR)**). Each dot represents an individual SNP. The slope of the regression line indicates the causal effect estimate. Positive slopes (**OR > 1**) suggest risk factors, while negative slopes (**OR < 1**) suggest protective factors. (A) IgD− CD38dim %lymphocyte on B cell on bladder cancer (B) IgD on unswitched memory B cells on bladder cancer (C) FSC-A on granulocyte on bladder cancer (D) HLA DR+ CD8br AC on TBNK on bladder cancer (E)FSC (forward scatter) (F)AC (absolute count) (G)TBNK (T-cell, B-cell, NK-cell)

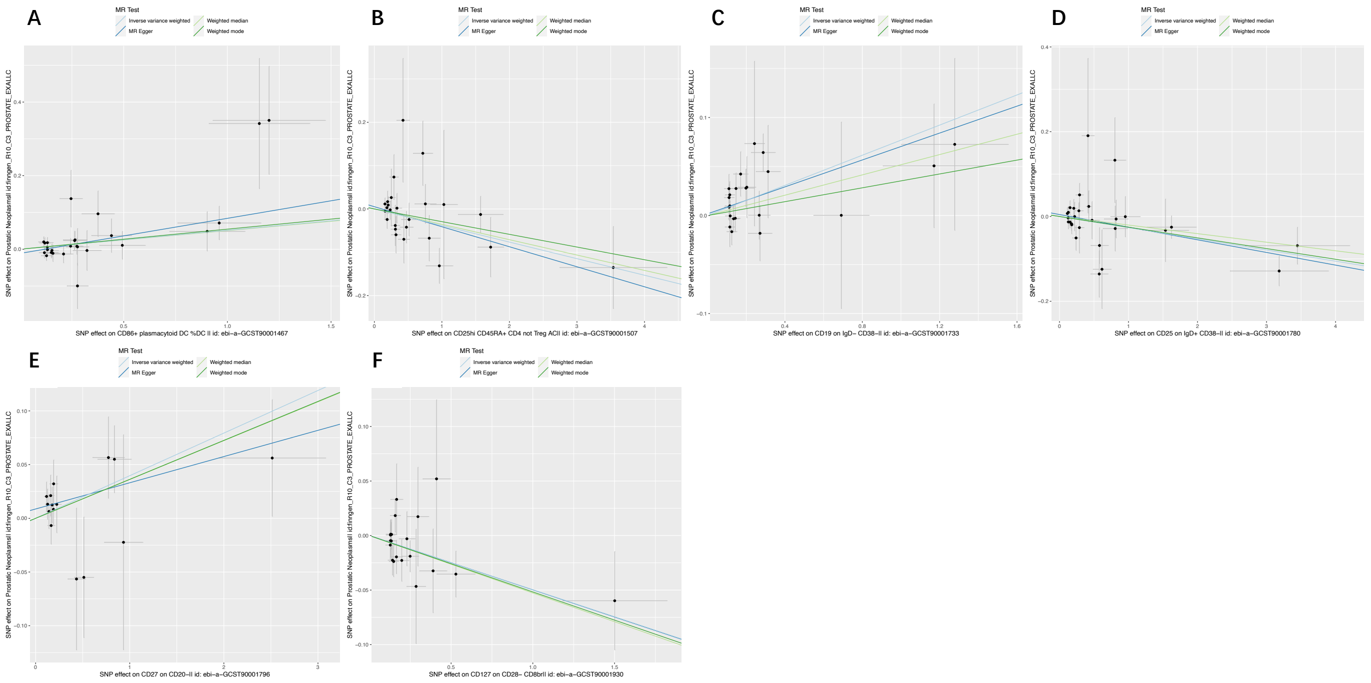

S3 Fig. Scatter plots of immune cell markers in prostate cancer This figure illustrates the association between immune cell markers and prostate cancer. The SNP effect sizes and their influence on prostate cancer risk were evaluated using **Inverse variance weighted (IVW)**, **MR Egger**, **weighted median**, and **weighted mode** methods. **Panels A to F**: The x-axis represents SNP effect sizes on immune cell markers, while the y-axis represents SNP effect sizes on prostate cancer risk (**log(OR)**). Each dot represents an individual SNP. The slope of the regression line reflects the causal effect estimate. Positive slopes suggest risk factors (**OR > 1**), while negative slopes suggest protective factors (**OR < 1**). (A) CD86+ plasmacytoid DC %DC on prostate cancer (B) CD25hi CD45RA+ CD4 T cell not Treg AC on prostate cancer (C) CD19 on IgD−CD38− B cell on prostate cancer (D) CD25 on IgD+ CD38− on B cell on prostate cancer (E) CD27 on CD20− on B cell on prostate cancer (F) CD127 on CD28− CD8br Treg on prostate cancer (G)AC(absolute count) (H)Treg(regulatory T cell) (I)DC(dendritic cell)

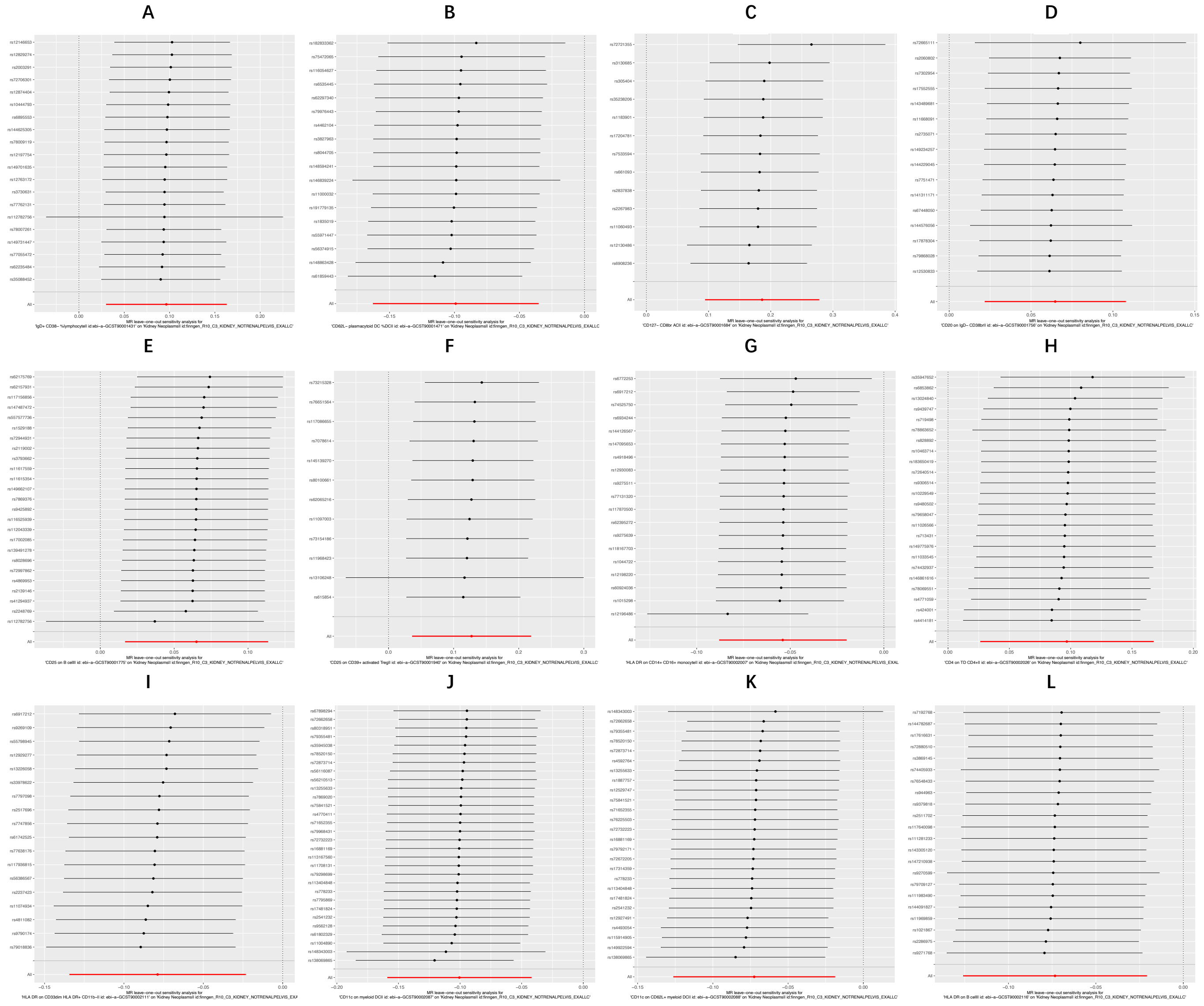

S4 Fig. Leave-one-out sensitivity analysis of immune markers in renal cancer This figure shows leave-one-out sensitivity analyses for the association between immune markers and renal cancer risk. Each panel represents a specific immune marker. **Panels A to L:** The x-axis shows the recalculated odds ratios (ORs) after removing one SNP at a time, while the y-axis lists the SNPs used as instrumental variables. Each line demonstrates the changes in ORs when a particular SNP is excluded, testing the robustness of the causal estimate. (A) IgD+CD38−%lymphocyte on B cell on renal cancer (B) CD62L− plasmacytoid Dendritic Cells on renal cancer (C) CD127− CD8br absolute count on renal cancer (D) CD20 on IgD− CD38br on B cell on renal cancer (E) CD25 on B cell on renal cancer; (F) CD25 on CD39+ activated Treg on renal cancer; (G) HLA DR on CD14+ CD16+ monocyte on renal cancer (H) CD4 on TD(terminally differentiated) CD4+ T cell on renal cancer (I) CD11c on myeloid DC (Dendritic Cell) on renal cancer (J) CD11c on CD62L+ myeloid dendritic cell on renal cancer (K) HLA DR on CD33dim HLA DR+ CD11b− Myeloid cell on renal cancer (L) HLA DR on B cell on renal cancer

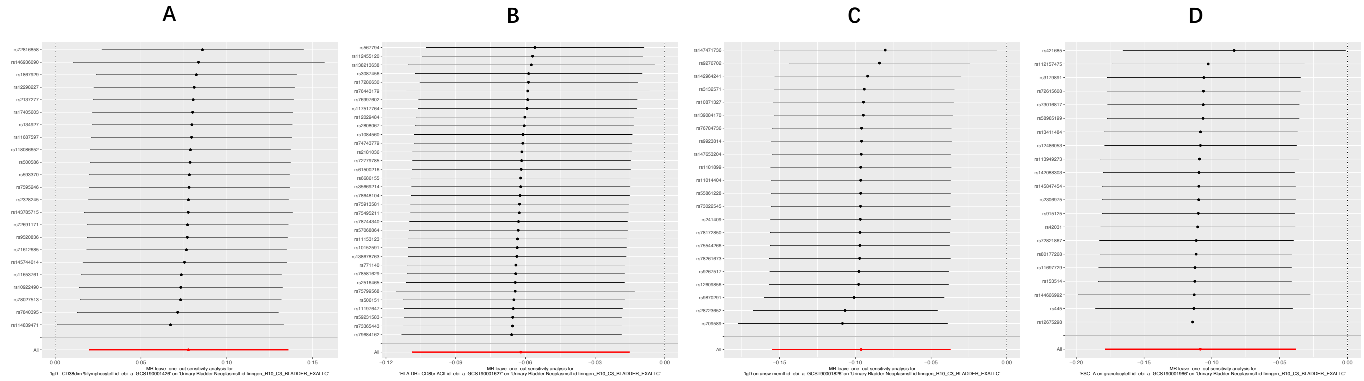

S5_fig. Leave-one-out sensitivity analysis of immune markers in bladder cancer This figure provides leave-one-out sensitivity analyses for immune cell markers and their relationship with bladder cancer. Each panel represents a specific immune marker. **Panels A to D**: Each panel represents a specific immune marker. The x-axis shows the recalculated odds ratios (ORs) after removing one SNP at a time, while the y-axis lists the SNPs used as instrumental variables. Each line demonstrates the changes in ORs when a particular SNP is excluded, testing the robustness of the causal estimate. (A) IgD−CD38dim %lymphocyte on B cell on bladder cancer (B) IgD on unswitched memory B cells on bladder cancer (C) FSC-A on granulocyte on bladder cancer (D) HLA DR+ CD8br AC on TBNK on bladder cancer

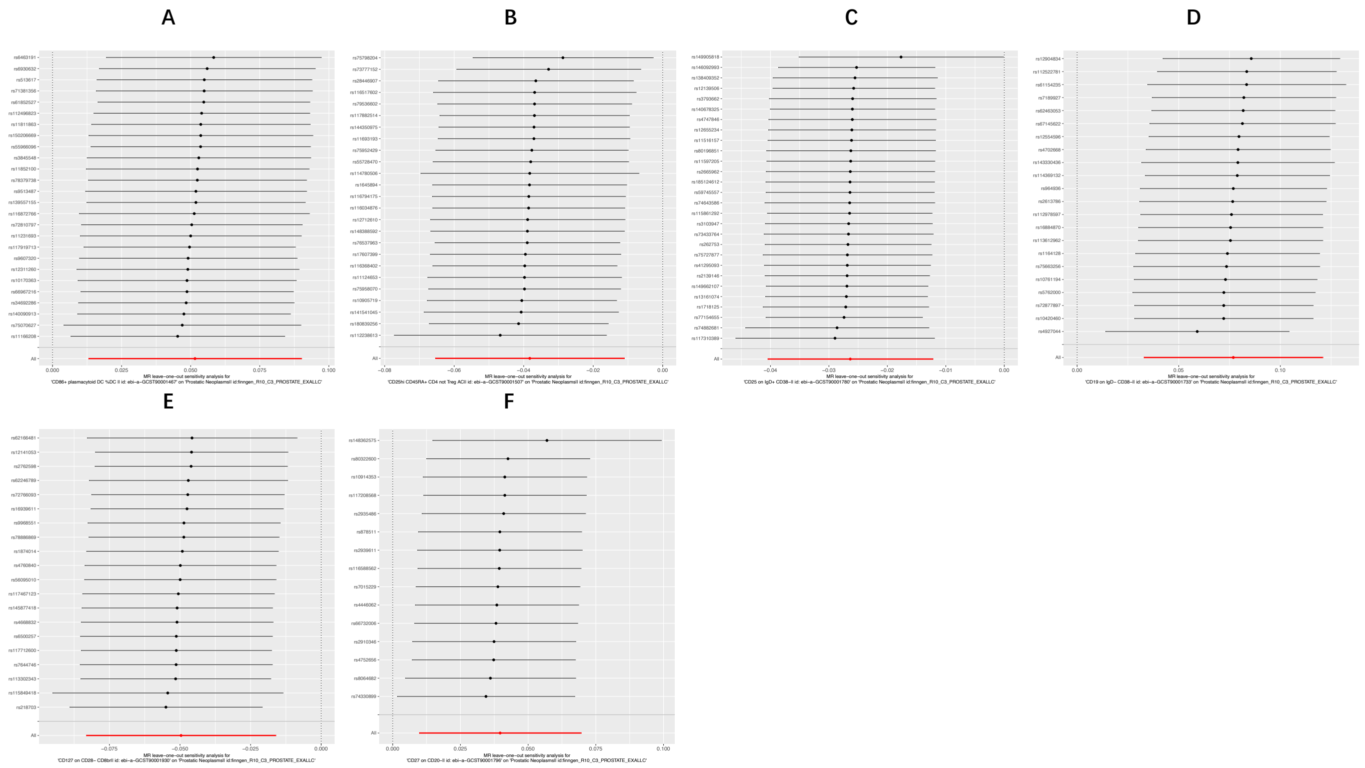

S6_fig. Leave-one-out sensitivity analysis of immune markers in prostate cancer This figure demonstrates leave-one-out sensitivity analyses for immune markers and prostate cancer risk. Each panel represents a specific immune marker. **Panels A to F**: The x-axis shows the recalculated odds ratios (ORs) after removing one SNP at a time, while the y-axis lists the SNPs used as instrumental variables. Each line demonstrates the changes in ORs when a particular SNP is excluded, ensuring the robustness of the findings. (A) CD86+ plasmacytoid DC %DC on prostate cancer (B) CD25hi CD45RA+ CD4 T cell not Treg AC on prostate cancer (C) CD19 on IgD− CD38− B cell on prostate cancer (D) CD25 on IgD+ CD38− on B cell on prostate cancer (E) CD27 on CD20− on B cell on prostate cancer (F) CD127 on CD28− CD8br Treg on prostate cancer
